# Scoping review of biological treatment of deficiency of interleukin-36 receptor antagonist (DITRA) in children and adolescents

**DOI:** 10.1186/s12969-019-0338-1

**Published:** 2019-07-08

**Authors:** Toni Hospach, Fabian Glowatzki, Friederike Blankenburg, Dennis Conzelmann, Christian Stirnkorb, Chris Sandra Müllerschön, Peter von den Driesch, Lisa Maria Köhler, Meino Rohlfs, Christoph Klein, Fabian Hauck

**Affiliations:** 1 Department of Pediatric Rheumatology, Klinikum Stuttgart, Olgahospital Stuttgart, Stuttgart, Germany; 2 Department of Dermatology, Klinikum Stuttgart, Bad Cannstatt, Stuttgart, Germany; 3 Department of Pediatrics, Dr. von Hauner Children‘s Hospital, University Hospital, LMU, Munich, Germany

**Keywords:** Generalized pustular psoriasis (GPP), IL36RN, Autoinflammation, DITRA, Deficiency of interleukin-36 receptor antagonist, Monogenic disease, Biologicals, Adalimumab

## Abstract

**Background:**

Deficiency of interleukin-36 receptor antagonist (DITRA) is a life threatening monogenic autoinflammatory disease caused by loss of function mutations in the *IL36RN* gene. Affected patients develop recurrent episodes of generalized pustular psoriasis (GPP) with systemic inflammation and fever. We here review and analyze the literature on pediatric DITRA patients who have been treated by biologicals targeting inflammatory cytokines.

**Method:**

A database research was performed to identify all relevant articles on pediatric DITRA patients treated with biologicals. According to defined response criteria therapeutic efficacy was analyzed.

**Results:**

Our literature research revealed 12 pediatric patients with DITRA who have received treatment with biologicals and we add a further not yet reported patient. Out of these 13 patients 10 were homozygous including 6 with the p.Leu27Pro, 3 with the p.Arg10 Argfs* and 1 with the p.Thr123Met mutation. 3 patients were compound heterozygous. In total 28 flares were treated with biological agents- targeting IL-1, IL-17, IL-12/23 and TNF-α. Complete response was achieved in 16 flares (57%), a partial reponse was seen in 2 flares (7%), and no response was observed in 10 flares (36%). Response rates were heterogeneous among the different agents. While complete/partial/no response with inhibition of TNF-alpha could be achieved in 7 (58%)/1 (8%)/4 (33%), the inhibition of IL-17 and of IL-12/23 led in each 4 flares to a 100% complete response. IL-1 inhibition led to complete/partial response in each 1 (13%) and was not effective in 6 (76%) flares. Of note, the novel patient was successfully treated with weekly dosed adalimumab.

**Conclusions:**

DITRA is a rare disease that has to be considered in GPP with systemic inflammation and fever. It can be effectively treated with specific biological inhibition of TNF-alpha, IL-12/23 and IL- 17, while anti-IL-1 treatment seems less effective. Weekly dosed adalimumab appears to be a treatment option for pediatric patients. Further reports and studies of biological treated pediatric DITRA patients are warranted for evaluation of optimal treatment.

## Background

Deficiency of interleukin-36 receptor antagonist (DITRA) is a life-threatening autoinflammatory disease caused by autosomal-recessive mutations in the interleukin-36 receptor *(L36RN* gene, OMIM 614204). Interleukin-36 receptor antagonist (IL-36RA) is an IL-1 family member that antagonizes proinflammatory signals of the IL-36 family members (IL-36α, IL36β and IL36γ) [9, 12, 36]. The major pathogenic role leading to skin and systemic inflammation is the impairment of the processing and regulatory function of IL-36RA [1]. Affected patients suffer from recurrent episodes of generalized pustular psoriasis (GPP) with systemic inflammation and fever [22]. Among the patients with GPP it was shown that mutations in the *IL36RN* gene, leading to DITRA, account for 21 to 82% of the disease [2, 16, 18, 23, 33]. Clinically, DITRA is different from GPP. DITRA patients have a younger age at onset and a higher risk of systemic inflammation [2, 16, 39]. DITRA is a rare disease, most often presenting in early childhood or adolescence. So far, no more than 200 patients have been reported – most of them from Asia [16, 21, 34, 38–40].

Treatment guidelines for adult and pediatric GPP do exist but are not available for DITRA [30]. Acitretin (ACI) has efficacy in inducing temporary remission. However, relapses are common and therapy for patients with refractory courses remains a clinical challenge [20, 39]. A recently published review on adult GPP showed the efficacy of targeted immunotherapy, e.g. that of TNF-α, IL-17, IL-12/23 and IL-1 blocking agents [2]. Therefore, the aim of our literature research was to summarize the data on pediatric DITRA patients treated with biologicals.

Furthermore we add our experience of one novel patient who was successfully treated with weekly dosed adalimumab, a treatment that was -to the best of our knowledge- not reported before in pediatric DITRA.

## Method

For scoping research we entered the terms “Deficiency of interleukin-36 receptor antagonist”, “Deficiency of interleukin-36 antagonist”, “IL36RN mutation” and “DITRA” into the NCBI pubmed, EMBO, Scielo, LIVIVO, and sciencedirect databases to identify all relevant articles from its inception to October 2018. The language was limited to English and German. Articles with disease age at onset over 18 years and those without biological treatment were excluded.

As there are no standardized response criteria for DITRA available we defined response as follows:

1. Complete response (CR) was defined as the absence of disease associated skin manifestations for at least one month. In this category patients were grouped reported to be in “total remission”, “complete remission”, “good response”, “free of skin eruptions”, “completely cleared skin”.
2. Partial response (PR) was defined as improvement -but still presence- of disease associated skin manifestations. In this category patients were grouped reported to be in “partial remission or partial response”, “positive effect, but not in total remission”, “no significant response”.
3. No response (NR) was defined as lack of improvement or deterioration of disease associated skin manifestation. In this category patients were grouped reported to have “no response”, “uncontrolled disease”, “no improvement” or “failure to treatment”.
4. A flare was defined as disease associated skin manifestation necessitating treatment change. Mild relapses were not defined as flare.
5. A mild relapse was defined as exacerbation that did either not require treatment, could be controlled with local treatment, with acitretin or only with dose modification of the already used biological.

**Fig. 1:**
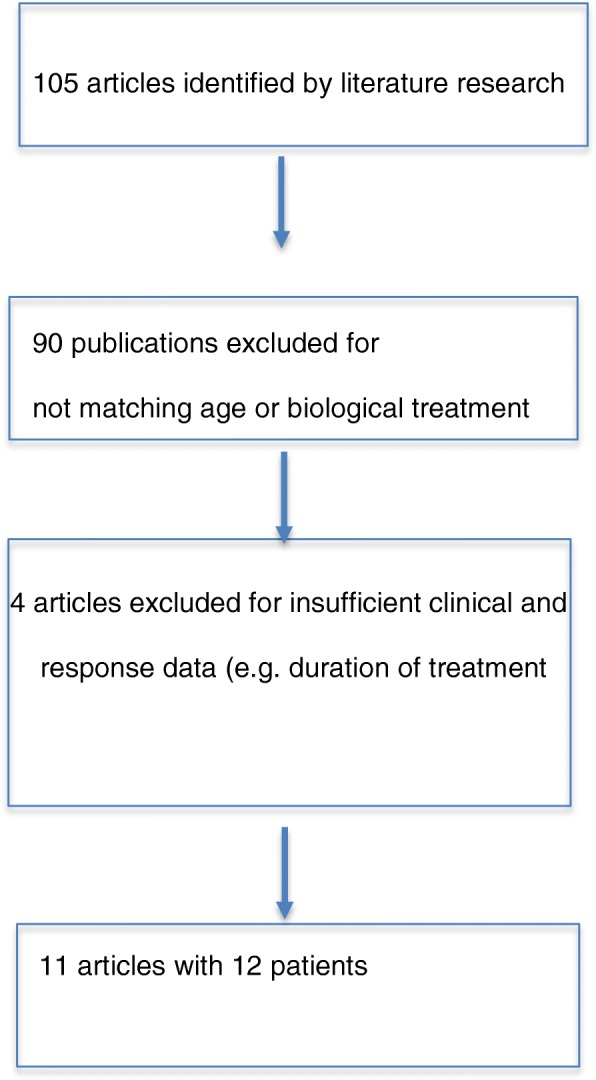
Flow chart of literature research

We identified 105 articles with the above mentioned search terms and excluded 90 that did not meet the inclusion criteria with age at onset below 18 years and/or biological treatment. Four articles were excluded for insufficient clinical and response data (e.g. duration of treatment). Of the remaining 11 articles all but one were single case reports [3] Fig. 1.

Informed consent was obtained of both parents in written and of the pediatric patient in oral form for publishing the case report and the pictures.

### Case report

**Fig. 2:**
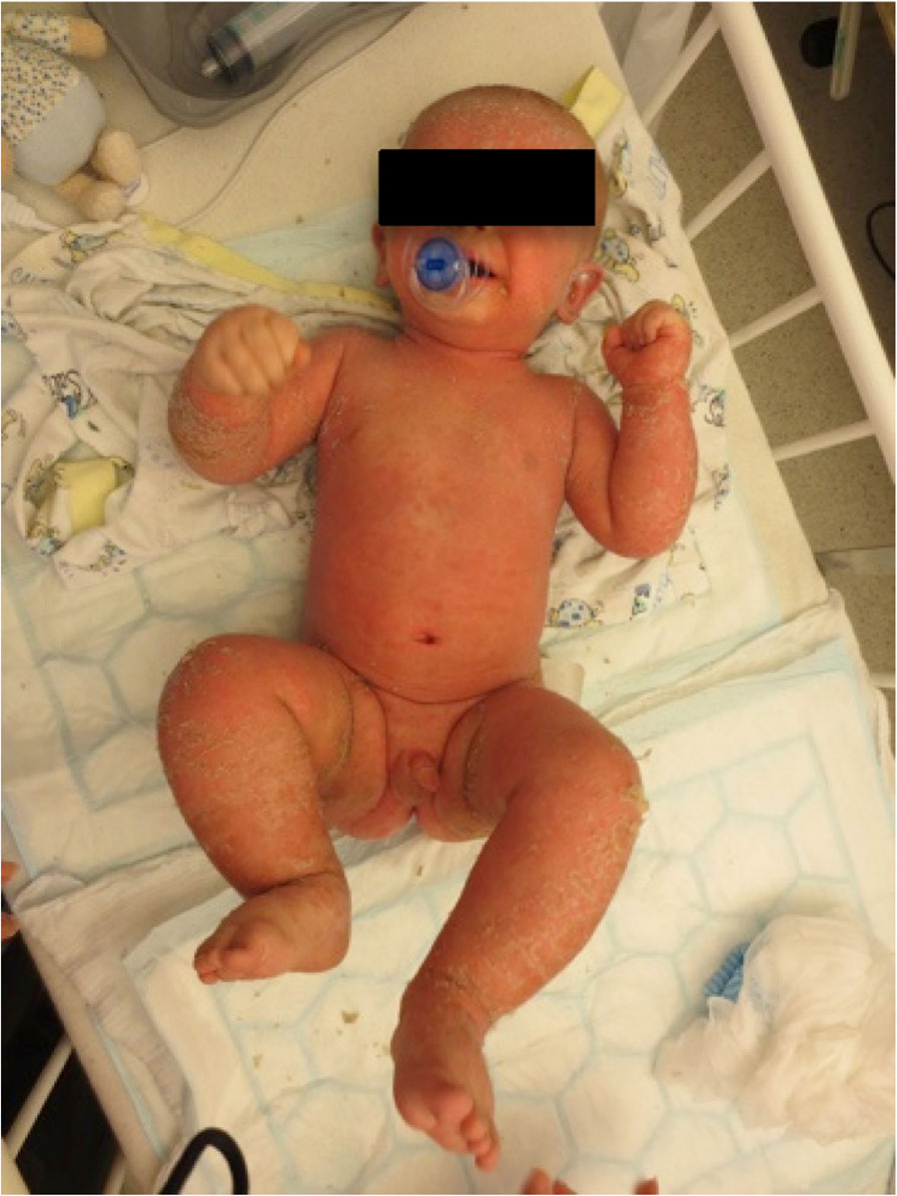
Generalized psoriatic lesions at the age of 7 month

**Fig. 3:**
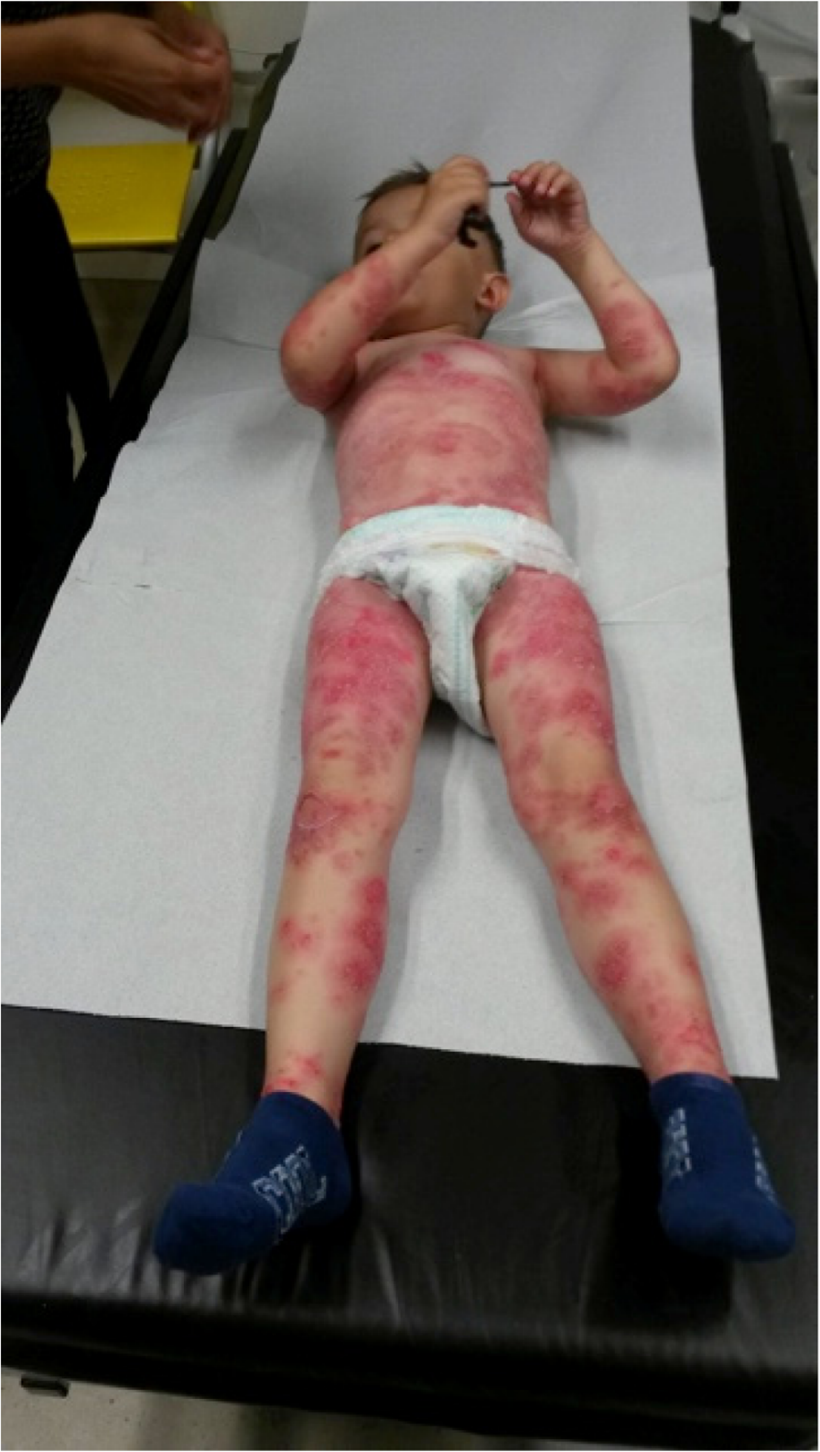
Generalized psoriasis at the age of 4 years

**Fig. 4:**
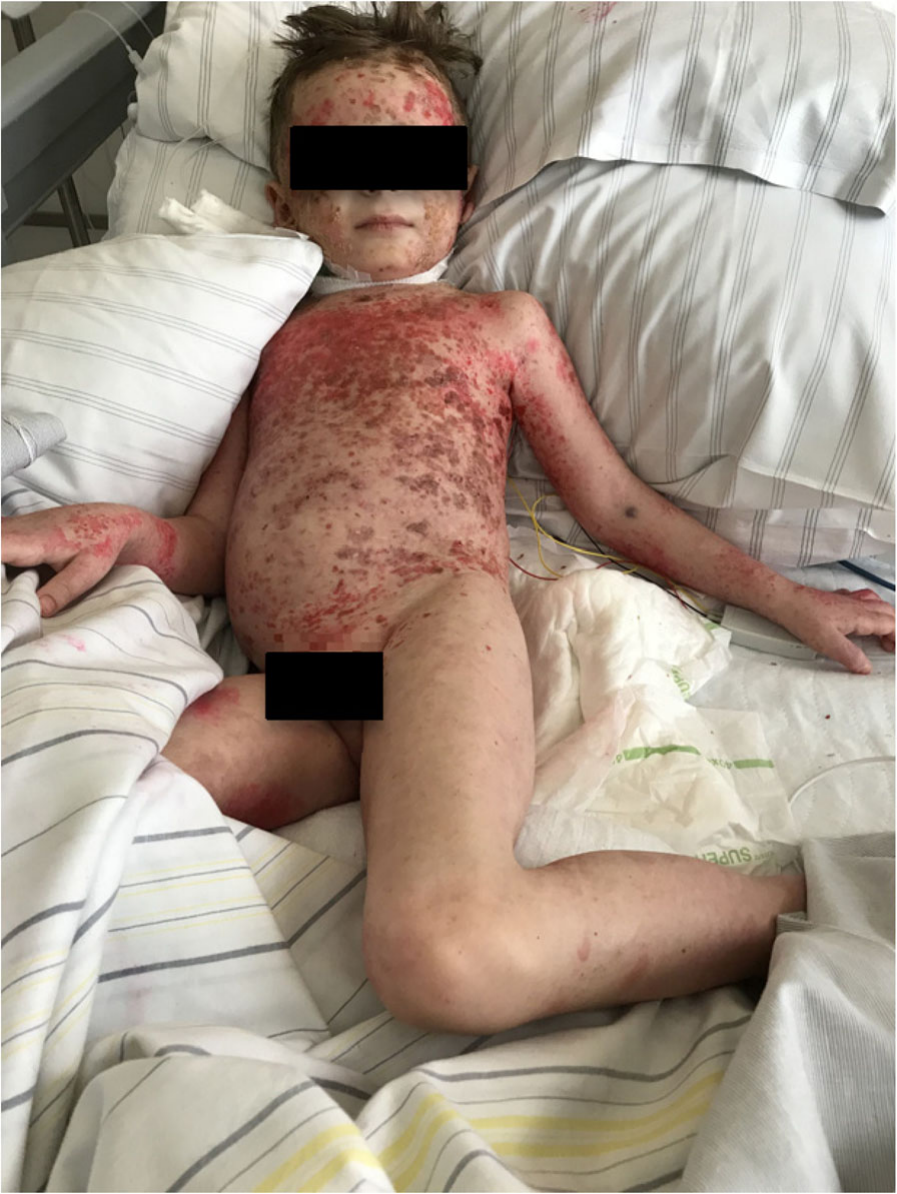
Psoriatic and pustular lesions before current treatment with adalimumab at age of 5 years

**Fig. 5:**
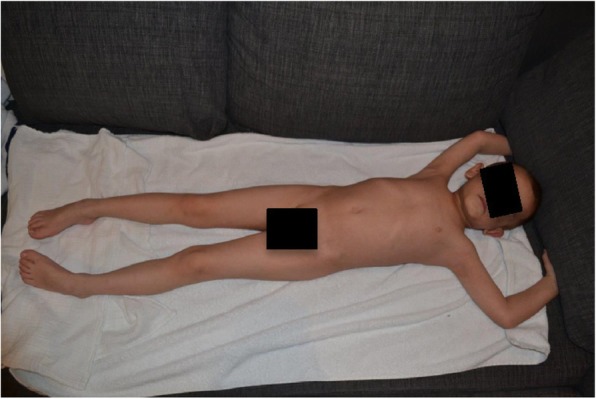
Complete resolution after treatment with adalimumab in weekly intervals

We report on a 5-year-old boy with recurrent severe acute exacerbations of generalized pustular psoriasis with fever. Starting at the age of 7 months (Fig. 2) he suffered from fever and psoriatic lesions. Diagnosis was “early onset psoriasis with probable infection”, although a causing infectious agent could not be recovered. Under initial treatment with antibiotics and with weekly methotrexate (MTX, 10 mg/sqm) and varying doses of prednisolone he responded completely over the course of several weeks. At the age of three years MTX could be stoppend. At the age of 4 years (Fig. 3) he again presented with fever and generalized psoriasis, being diagnosed as “generalized psoriasis”. With high doses of prednisolone (2 mg/kg), MTX (10 mg/sqm) and topical treatment he showed a partial response with recurrent lesions on the trunk. While prednisolone was tapered during the following weeks MTX was continued. The latest admission at the age of 5 years was necessary for severe pustular and psoriatic skin lesions covering 60% of his skin (Fig. 4). He had fever and CRP was elevated up to 11,8 mg/dl. Abdominal ultrasound revealed hepatosplenomegaly, thickened bileducts and ascites. Echocardiography revealed pericardial and pleural effusion. Lipase and y-GT were elevated to 2480 U/l and 213 U/l, respectively; ALT and AST were in normal ranges. Diagnosis then was “generalized pustular psoriasis”. We started methylprednisolone pulses (20 mg/kg) for 3 days and continued with prednisolone 2 mg/kg/d -additive to methotrexate 10 mg/sqm with minor efficacy related to the skin. After introduction of adalimumab 20 mg (body weight 16 kg) every week the skin cleared completely within 2 weeks, with normal temperatures and CRP values as well as yGT and lipase. Echocardiography and ultrasound of the abdomen normalized. After 12 months the boy is in complete remission with adalimumab in weekly intervals and MTX 10 mg/sqm (Fig. 5).

**Fig. 6:**
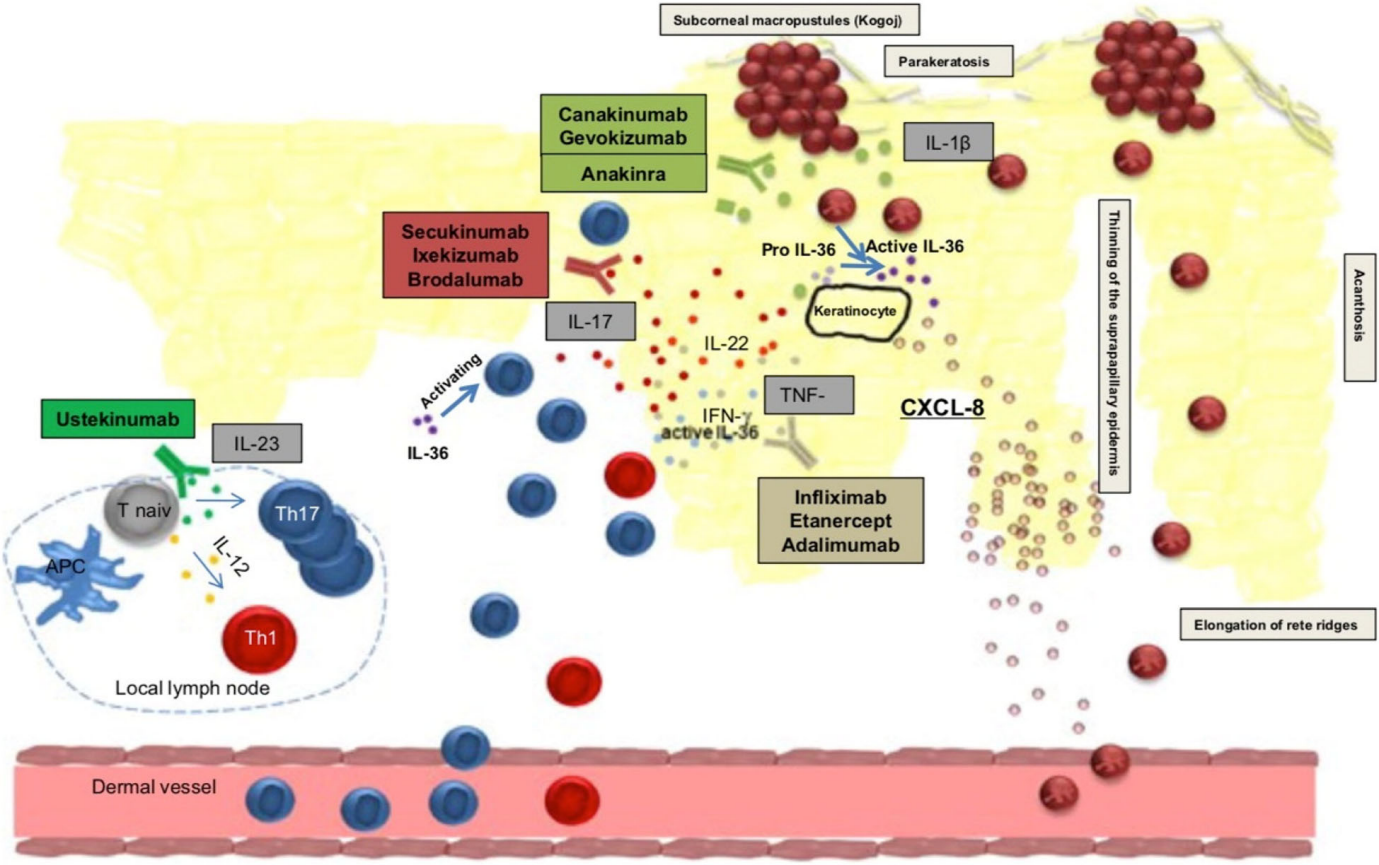
Pathogenesis of GPP (main mediators) including targets for immunotherapy [2] (reprinted with permission)

To establish a definitive diagnosis, exom sequencing was performed on the patient and his parents. Initially, we assumed consanguinity and filtered accordingly for a homozygous *STXBP2* variant (c.568C > T; p.Arg190Cys). Pathogenic autosomal recessive *STXBP2* variants can lead to familial hemophagocytic lymphohistiocytosis type 5 (FHL5) and the systemic inflammatory response observed in the patient could have indeed been an atypical FHL5 manifestation [27]. However, functional testing of NK cell degranulation was normal and the variant was therefore considered benign [4]. Next, the exome data were re-analysed assuming non-consanguinity and two variants in the *IL36RN* gene were identified. Sanger sequencing of both parents and the patient confirmed compound heterozygous *IL36RN* variants (c.227C > T; p.Pro76Leu and c.338C > T; pSer113Leu). In light of the clinical phenotype, the genetic data is consistent is in line with establishing the diagnosis DITRA.

## Results of the literature research

Our literature research revealed 13 pediatric DITRA patients – including the one reported here - treated with biologicals (Table 1). All but one were pretreated with multiple nonbiological agents. Male to female ratio was 10:3. Median age at onset was 7 month (range 0.5–192 months), whereas median age at genetic diagnosis was 55 months (range 2–204 months) summing up to a diagnostic delay of 47 months. 10 patients were homozygous for a mutation in *IL36RN*, including 6 with the p.Leu27Pro mutation representing the most common mutation, 3 with the p.Arg10 Argfs* splice mutation and one with the p.Thr123Met mutation. Three patients were compound heterozygous (Table 1) for mutations in *IL36RN*. In total, 28 flares in 13 patients were treated with biological agents. Median treatment duration was 5.5 months (range 0,1–36 months). Concomitant treatment with DMARDs, ACI, IVIG or PRED was given in 6 flares in general, and in 4 flares with CR (low dose MTX and PRED, ACI and IVIG). Complete response was achieved in 57% of the flares. Partial response was seen in 7%, while no response was reported in 36% of the flares. Response rates were heterogeneous among the different agents. While complete/partial/no response with inhibition of TNF-alpha could be achieved in 58%/8%/33% of the flares, the inhibition of IL-17 and of IL-12/23 led to a 100% complete response. IL-1 inhibition led to complete/partial response in each 13% and was not effective in 76% of the flares. Follow-up intervals after cessation of treatment were only reported in two patients (Table 3, for details see Table 2).

**Table 1:** Patient characteristics of 13 pediatric DITRA patients treated with biological agents

Case	Age at onset/genetically confirmed diagnosis in months	sex	Mutation c (p) nomenclature	Zygosity	Previous treatment failures with nonbiological agents	Author
1	72/96	M	c.115 + 6 T > C (p.Arg10Argfs*)	ho	CSA, MPP, ACI	[28]
2	“infancy”/“adolescence”	M	c.115 + 6 T > C (p.Arg10Argfs*)	ho	ACI, CSA, PUVA, PRED, MTX, APRE	[8]
3	1/72	M	c.115 + 6 T > C (p.Arg10Argfs*)	ho	CS, MTX, ACI,CSA	[15]
4	0.5/6	M	c.80 T > C (p.Leu27Pro)	ho	–	[31]
5	2/36	M	c.80 T > C (p.Leu27Pro)	ho	Topical CS, systemic RET	[5]
6	41/48	M	c.80 T > C (p.Leu27Pro)	ho	CS, MTX	[19]
7	2/17	F	c.80 T > C (p.Leu27Pro)	ho	CSA, ACI	[10]
8	1/2	M	c. 80 T > C (p.Leu27Pro)	ho	Topical CS, ACI	[6]
9	1/60	F	c. 80 T > C (p.Leu27Pro)	ho	CS, ACI, CSA	[3]
10	NA/"adolescence"	M	c.368C > T (p.Thr123Met)	ho	MTX, ACI	[24]
11	84/NA	F	c.142C > T/c.338C > T (pArg48Trp)/p.Ser113Leu)	comp het	PRED, ACI, ISO, MTX, CSA	[26]
12	7/60	M	c.227C > T/c.338C > T (p.Pro76Leu)/(pSer113Leu)	comp het	MTX, CS	Patient reported here
13	36/48	M	c.227C > T/c.338C > T (p.Pro76Leu/p.Ser113Leu)	comp het	CS, CSA, THAL, DAP	[3]

Abbr.:ACI-acitretin, APRE-apremilast, CS-corticosteroids, CSA-ciclosporine, DAP-dapsone, ho-homozygosity, het-heterozygosity, comp het-compound heterzygosity, ISO-isotretinoin MPP-methylprednisolone, MTX-methotrexate, NA-not available, PRED-prednisolone, PUVA-psoralen plus UVA, RET-retionoids, THAL-thalidomide.

**Table 2:** Detailed overview of treatment with biological agents in 28 flares of 13 pediatric patients

Target	Drug	Dose	Pustule cleareance (day)	Clinical improvement number of patients/total number treated	Clinical response type	Concomitant systemic treatment	Treatment duration (months)	Reference
TNF-a	ETA	NA	NA	1/1	PR	MTX	4	[19]
		0.4 mg/kg twice per wk	NA	1/1	CR*	NA	7	[15]
		12.5 mg/wk	35	1/1	CR	ACI 1 mg/kg/d	6	[10]
		NA	NA	0/1	NR	ACI	7	[3]
		0.8 mg/kg/wk	NA	0/1	NR	–	3	[6]
	ADA	20 mg /every 2 wk	NA	0/1	NR	NA	NA	[15]
		NA	NA	1/1	CR**	NA	26	[8]
		NA	NA	0/1	NR	NA	1	[3]
		20 mg every wk	7	1/1	CR	–	8	Patient reported here
	IFL	100 mg in 2 wk. interval, in total 2 infusions (3,3 mg/kg)	14	1/1	CR**	-	0,5***	[28]
		5 mg/kg	NA	1/1	CR**	NA	9	[8]
		NA	NA	1/1	CR	NA	1	[26]
IL-1	ANA	4 mg/kg/d	7	1/1	CR	–	2	[31]
		5 mg/kg/d	NA	0/1	NR	NA	3	[5]
		100 mg/d	NA	0/1	NR	NA	3 days	[8]
		6 mg/kg/d	NA	0/1	NR	NA	NA	[19]
		5 mg/kg/d	NA	0/1	NR	NA	NA	[10]
		NA	NA	0/2	NR	NA	2/0,2	[3]
		6 mg/kg/d	Partial	1/1	PR	–	36	[6]
	CAN	3 mg/kg/d every 8 wks	NA	0/1	NR	NA	NA	[5]
IL12/23	UST	NA	NA	1/1	CR*	NA	31	[8]
		Pat 1: 1.5 mg/kg every 2 mths Pat 2: 1 mg/kg every 2 mths	NA “weeks”	2/2	CR CR	IVIG-	155	[3]
		0.75 mg/kg every 3mths	NA	1/1	CR	–	12	[6]
IL-17	SEC	150 mg/wkly ervery 4 wks	1	1/1	CR	MTX 5 mg/wk	12	[8]
		75 mg/wk	NA	1/1	CR**	PRED 2.5 mg/d	2	[19]
		150 mg mthly	14	1/1	CR**	–	8****	[15]
		300 mg every 4 wks, initially 5 doses 300 mg wkly	2	1/1	CR**	–	6	[24]

Abbr. ADA-adalimumab, ANA-anakinra, CANA-canakinumab, CR-complete response, ETA-etanercept, IFL-infliximab, MP-methylprednisolone, NA-not available, NR-no response, PRED-prednisolone, PR-partial response, SEC-Secukinumab, UST-ustekinumab, * secondary failure after 7 months, changed to SEC **mild relaps, *** 21 months of follow-up interval after cessation of treatment, **** 4 months of follow-up interval after cessation of treatment.

## Discussion

In pediatric patients with systemic inflammation and GPP monogenic diseases like deficiency of interleukin 1 receptor antagonist (DIRA), DITRA and caspase activation and recruitment domain (CARD) 14-mediated psoriasis (CAMPS) have to be considered [13, 25]. Our study highlights a marked delay of 47 months between age at onset and genetically confirmed diagnosis. Thus, clinical awareness should be raised in order to rapidly initiate genetic testing and effective treatment. In these diagnostic procedures close collaboration with experts in clinical immunology, rheumatology, dermatology, and molecular genetics is advisable for correct interpretation of the plethora of polymorphisms and identification of pathological mutations. Our clinical case illustrates these challenges. A homozygous variant in the *STXBP2* gene (c.568C > T; p.Arg190Cys), initially interpreted as possible cause for atypical FHL5, could eventually be excluded through repetitive interdisciplinary discussions, in-depth functional immune assays, and re-interpretation of exome sequencing data – testifying to the value of good interdisciplinary collaboration in the best interest of patients.

Whereas the pathogenicity of the *IL36RN* mutations in the patient remains to be formally proven, both compound heterozygous *IL36RN* mutations have been reported in DITRA patients before. The mutation c.227CY > T; p.Pro76Leu has been described as an amorphic variant, the mutation c.338C > T; pSer113Leu was considered to be hypomorphic [34]. This compound heterozygous mutation has also been reported in another patient [3]. To the best of our knowledge associations of FHL5 and IL36RN gene variants have not been reported so far.

Early treatment of GPP is essential to prevent bacterial superinfection and probably death that was estimated to occur in 4–7% [2, 7, 17]. Treatment recommendations for GPP in children do exist from the American Board of National Psoriasis Foundation and include acitretin, cyclosporine A, methotrexate and etanercept as first line treatment and adalimumab, infliximab and ultraviolet B phototherapy as second line treatment [30]. As no guidelines exist for therapy of DITRA and a recent report on targeted immunotherapy in GPP exclusively addresses adults it is important to collect data on the efficacy of biological treatment for children. The rational for treatment with biological agents is given through significant overexpression of Il-17A, TNF-α, IL-1, IL-36 in GPP and in psoriasis (see Fig. 6).

Our literature research showed that biologically treated DITRA flares resulted in 57% in complete response. This is comparable to the data on 101 adult cases with GPP -including four DITRA patients- where 61% showed complete response [2]. This is also true for the response rates to TNF-alpha blocking agents. Each 58% in pediatric and adult GPP patients showed a complete response [2]. While there is evidence that autoinflammation has a role in the pathogenesis of psoriasis and GPP [11, 29] treatment responses to IL-1 blocking agents were lowest in our review (Table 3). This is especially true for the reported doses of Anakinra of maximally 6 mg/kg. Interestingly, adult GPP patients with IL-1 treatments also only showed a 20% rate of complete response [2, 37]. It was hypothesized that the inefficacy of anti-IL-1 treatment might be due to the yet not clear activity of IL-1 downstream of IL-36 [35]. Inhibition to IL-12/23 blocking agents (ustekinumab) was successful in all four patients (Tables 2,3). This biologic agent has before been shown to be effective in two pediatric DITRA patients. Nevertheless, high doses were required to achieve complete response [3]. Meanwhile a recently published case report also showed efficacy with standard dosing [6]. In adult GPP patients six out seven ustekinumab treated flares had complete response [2]. Treatment with IL-17 inhibition (secukinumab) also showed a complete response in all four flares (Tables 2,3) and even as monotherapy in two of them [15, 24]. In 32 adults complete remission was achieved in 76% and partial response 27% with this substance [2]. Taken into account that with either IL-12/23 and with Il-17 inhibition mild relapses occurred, these two substances have to be considered as an effective option in pediatric DITRA. In addition patients may also benefit from our approach of weekly administered adalimumab which has -to the best of our knowledge- not been reported in pediatric DITRA before.

**Table 3:** Outcome of biological treatment in 28 flares of 13 pediatric patients

Targets	Flares n	Clinical response [flares n (%)]			Median treatment duration in months (range)
		CR	PR	NR	
TNF-a	12	7 (58)	1 (8)	4 (33)	6.5 (0.2–26)
IL-1	8	1 (13)	1 (13)	6 (76)	2.5 (0.1–36)
IL-12/23	4	4 (100)	–	–	13.5 (5–31)
IL-17	4	4 (100)	–	–	8 (2–12)
Total	28	16 (57)	2 (7)	10 (36)	5.5 (0.1–36)

Abbr. CR-complete response, PR-partial response, NR-no response.

The reason for the heterogeneous response to biologicals is unclear, although a correlation between the severity of the disease and the degree of the functional impairment of the mutated IL36RN could be shown [34]. Besides this proposed phenotype-genotype association with severe clinical manifestation in patients with nonsense mutation further factors seem to play a role as reported by several authors who showed that similar treatments in patients with identical mutations resulted in different outcome [5, 10, 28, 31, 32]. Our review did not show an association of treatment response with genetic mutation either, be it because of the small number of the cohort or because there is no direct one (data not shown).

Limitations of our scoping that may lead to bias could result from lacking data on long-term observation as follow-up intervals after cessation of treatment were only reported in two flares. Furthermore our defined remission period of one month is relatively short. This restriction was necessary due to four flares reporting CR with shorter observation periods than 6 months. These limitations might be important as the natural course of the disease is characterized by changing patterns of activity. Secondly the role of concomitant treatment in four flares with CR (low dose MTX and PRED, ACI and IVIG) can not be clearly evaluated. It is obvious that a retrospective data collection on a small cohort of patients is prone to bias through relative predominance of singular events. On the other hand, regarding the scarcity of the published cases and the need to introduce effective treatments it seems necessary to analyze the existing data.

In summary we have reviewed the literature on pediatric DITRA patients that have been treated with biologicals. Good response was seen with TNF blocking agents and especially with inhibition of IL-17 and IL-12/23, while anti-IL treatment was less effective. For accurate evaluation of the efficacy of these substances-also in respect to induction and maintenance treatment- long-term observation is necessary. Although clincal trials with biologicals in GPP are under way- including adalimumab in japanese adolescents- use of these agents in pediatric patients with DITRA is off-label (clinicaltrials.gov). If the recently developed anti-human and anti-mouse IL-36R antagonist monoclonal antibodies will have clinical effects for DITRA patients has to be proven [14].

## Conclusion

DITRA is a rare disease that has to be considered in GPP with systemic inflammation and fever. For diagnostic purposes close collaboration between clinicians and geneticists is important. DITRA can be effectively treated with specific biological inhibition of TNF-alpha, IL-12/23 and IL- 17, while IL-1 treatment seems less effective. Further reports and studies of biological treated pediatric DITRA patients are warranted for evaluation of optimal treatment.

## Abbreviations

ACI: Acitretin
ADA: Adalimumab
ANA: Anakinra
APRE: Apremilast
CAMPS: Caspase activation and recruitment domain 14-mediated psoriasis
CANA: Canakinumab
CARD: Caspase activation and recruitment domain
comp het: Compound heterzygosity
CR: Complete response
CRB: Complete response with breakthrough
CS: Corticosteroids
CSA: Ciclosporine
DAP: Dapsone
DITRA: Deficiency of interleukin-36 receptor antagonist
ETA: Etanercept
FHL5: Hemophagocytic lymphohistiocytosis type 5
GPP: Generalized pustular psoriasis
Het: Heterozygosity
Ho: Homozygosity
IFL: Infliximab
IL-1: interleukin-1
IL-12/23: Interleukin-12/23
IL36RN: Interleukin-36 receptor antagonist
IL-17: Interleukin-12
ISO: Isotretinoin
MAPKs: Mitogen activated protein kinases
MPP: Methylprednisolone
MTX: Methotrexate
NA: Not available
NF-κB: Nuclear factor kappa B
NR: No response
PR: Partial response
PRED: Prednisolone
PUVA: Psoralen plus ultraviolet A
RET: Retionoids
SEC: Secukinumab
STXBP2: Syntaxin-binding protein 2
THAL: Thalidomide
TNF-α: Tumornecrosisfactor- α
UST: Ustekinumab
